# A systematic review of health status, health seeking behaviour and healthcare utilisation of low socioeconomic status populations in urban Singapore

**DOI:** 10.1186/s12939-018-0751-y

**Published:** 2018-04-02

**Authors:** Catherine Qiu Hua Chan, Kheng Hock Lee, Lian Leng Low

**Affiliations:** 1 Department of Family Medicine & Continuing Care, Singapore General Hospital, Singapore, Singapore; 2 Family Medicine, Duke-NUS Medical School, Singapore, Singapore; 3 Family Medicine Academic Clinical Program, SingHealth Duke-NUS, Singapore, Singapore

**Keywords:** Public rental housing, Low socioeconomic, Health status, Singapore

## Abstract

**Introduction:**

It is well-established that low socioeconomic status (SES) influences one’s health status, morbidity and mortality. Housing type has been used as an indicator of SES and social determinant of health in some studies. In Singapore, home ownership is among the highest in the world. Citizens who have no other housing options are offered heavily subsidised rental housings. Residents staying in such rental housings are characterised by low socioeconomic status. Our aim is to review studies on the association between staying in public rental housing in Singapore and health status.

**Methods:**

A PubMed and Scopus search was conducted in January 2017 to identify suitable articles published from 1 January 2000 to 31 January 2017. Only studies that were done on Singapore public rental housing communities were included for review. A total of 14 articles including 4 prospective studies, 8 cross-sectional studies and 2 retrospective cohort studies were obtained for the review. Topics addressed by these studies included: (1) Health status; (2) Health seeking behaviour; (3) Healthcare utilisation.

**Results:**

Staying in public rental housing was found to be associated with poorer health status and outcomes. They had lower participation in health screening, preferred alternative medicine practitioners to western-trained doctors for primary care, and had increased hospital utilisation. Several studies performed qualitative interviews to explore the causes of disparity and concern about cost was one of the common cited reason.

**Conclusion:**

Staying in public rental housing appears to be a risk marker of poorer health and this may have important public health implications. Understanding the causes of disparity will require more qualitative studies which in turn will guide interventions and the evaluation of their effectiveness in improving health outcome of this sub-population of patients.

## Background

Singapore is an urbanised Asian society with high cost of living. In a world-wide survey on the cost of living, Singapore emerged as the most expensive city to live in [1]. The affordability of housing is a major area of concern. In a survey of voters, cost of living and affordability of housing was among the top 3 issues that people worry about most. Income disparity in the country is high with a GINI Co-efficient of 0.458 in 2016 [2]. Even then, this was the lowest level in a decade after concerted government efforts to reverse a widening trend. At the same time, Singapore’s population is ageing rapidly, and 1 in 5 persons will be over 65 years old by the year 2030 [3]. Increased healthcare spending is anticipated and Singapore’s healthcare costs is expected to rise nearly tenfold over the next 15 years. With high levels of income disparity, increasing healthcare cost and a decreasing old-age support ratio, elderly persons with poor social support are at the highest risk of having poor health outcomes. These at-risk seniors are most likely to reside in public rental housing which are heavily subsidised by the Singapore government.

It is well-established that low socioeconomic status (SES) influences one’s health, rate of morbidity and mortality [4–6]. SES influence health via the interaction between the individual’s socioeconomic characteristics as well as their area’s socioeconomic conditions [7, 8].

Educational level, income status, employment status, financial assistance requirement are examples of individual-level measures of SES. Unfortunately, such information are not routinely collected during healthcare encounters. Hence, using such measures to identify at risk population may not be practical. On the other hand, housing information such as residency in public rental housings are readily available and has been used as an area-level measure of SES. This information is collected in almost all healthcare encounters and the unique postal codes correspond to individual houses and apartment blocks.

Housing has been known to be an important social determinant of health [9, 10]. In Singapore, housing is not geographically segregated according to SES, instead there is a mixture of public rental housing together with owner-occupied public housing within each residential precinct. The home ownership rate of resident households in Singapore is among the highest in the world at 90.9% [11]. Households that cannot afford to purchase their own homes and have no other housing options are offered heavily subsidized rental housings under the Public Rental Scheme [12]. Such rental housings form clusters within residential precincts and studying residents in these micro-communities may provide useful insights on the impact of low socioeconomic status on health, within a relatively affluent society.

Using residency in public rental housing as a marker of low SES, our aim is to review studies on the association between staying in public rental housing in Singapore (as a low SES community) and health (health status, health seeking behaviour and healthcare utilisation).

## Methods

### Search strategy

**Fig. 1:**
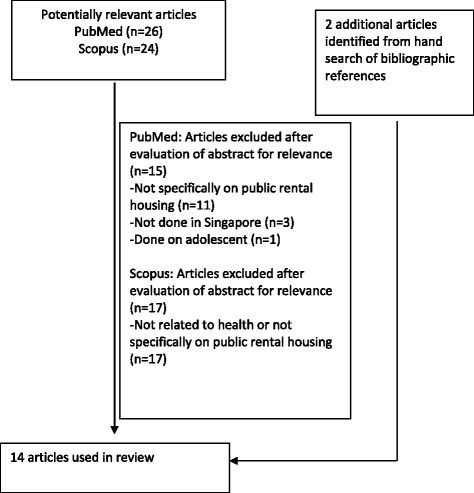
Flowchart on selection of articles

A PubMed and Scopus search was carried out in January 2017 to identify potentially relevant articles published from 1 January 2000 to 31 January 2017. The following search strategy was applied: “Socioeconomic” (MeSH term) AND “Housing” (MeSH term) AND “Singapore” (MeSH term) AND (“Health status” OR “Health-seeking behaviour” OR “Healthcare utilisation”). Hand search of bibliographic references of the shortlisted articles was also conducted. The search strategy was summarised in the following flow chart (Fig. 1).

### Inclusion and exclusion criteria

Two authors (C.Q.H Chan and L.L Low) independently reviewed the articles for inclusion and exclusion. We included only reviews, qualitative and experimental quasi-experimental research articles that studied Singapore public rental housing communities, were in English and investigated residents above 21 years of age. Articles were included if they contained information on health (health status, health-seeking behaviour, healthcare utilisation and perceptions of health / health services) related to residents staying in public rental housing in Singapore. We excluded editorial, perspective, commentary and expert opinion articles and also those that do not study health, or public rental housing residents specifically or are not done in Singapore.

### Quality assessment

The Newcastle-Ottawa Scale (NOS) was used to assess the quality of the articles. Out of 9 stars, 0–4 star(s) was/were considered low quality; 5–6 stars were considered for fair quality; 7–9 stars were considered good quality.

## Results

As shown in Figs. 1, 26 and 24 potentially relevant articles were retrieved through the PubMed and Scopus searches respectively. The abstracts of these articles were evaluated for relevance to the aims of this review. Articles that were not related to health, or not done specifically on public rental housing or not based in Singapore were excluded. Another 2 articles were identified from hand search of bibliographic references of the shortlisted articles. A total of 14 articles which includes 4 prospective studies [13–16], 8 cross-sectional studies [17–24] and 2 retrospective cohort studies [25, 26] were obtained for the review. Among these studies, 4 of the articles included qualitative interviews [14, 20, 23, 24]. Effect of a community interventional program on outcomes was also studied in 4 of the articles [13, 15, 16, 22]. Most of the prospective studies were done with the comparison of outcomes between the rental housing and owner-occupied housing community. Tables 1 and 2 summarised the description and results of the 14 articles reviewed.

**Table 1:** Description and the results of articles reviewed

Study (Year of publication), Study type, Duration of study	Research question	Demographics/ Intervention done	Results	Conclusion	Level of evidence (based on NOS)
Health status					
Wong TH (2017) [26] Retrospective cohort From 1992 to 2014	Survival of patients with head and neck squamous cell carcinoma (HNSCC).	Patients with HNSCC in National Cancer Center database. Mortality information matched from Singapore Death Registry. Non-residents were excluded from analysis.	Sample size 686 patients were analysed, 84 (11%) of them were rental housing residents. Primary outcome • All-cause mortality: Patients living in postal codes with a room index < 3.0* had the worst survival [median, 28 months, CI 21–48 months] compared to those staying in larger housing sizes (higher room index) and owner occupied. Secondary outcome Disease stage at presentation: Patients living in a lower room index postal code were not more likely to present with advanced disease. *majority of residents lived in rental housings with a monthly household income <S$1500	Patients with HNSCC living in smaller, higher-subsidy housings have poorer survival despite no apparent delays in presentation.	Poor
Wee LE (2013) [13] Prospective, Interventional From 2009 to 2011	Hypertension management and lifestyle changes following screening for hypertension.	Residents > 40 y/o. two public rental housing precincts. Site A (Western Singapore) and Site B (Eastern Singapore). Intervention: 6-month community-based intervention comprising access-enhanced screening component and follow-up (outreach) component.	Sample size 577 residents Participation rate 83.2% (577/693) Follow up rate (follow up for 1 year) 80.9% (467/557) Prevalence rate Baseline: 60.4% (282/467) Known hypertension *n* = 179, Newly diagnosed *n* = 103 Primary outcome Untreated and uncontrolled hypertension • Baseline: Known hypertension and not treated *n* = 48 Untreated hypertension 53.5% (151/282) Known hypertension and treated *n* = 131 Uncontrolled hypertension despite treated 54.2% (71/131) • Post intervention: Untreated hypertension 48.3% (73/151) Started treatment *n* = 78 Uncontrolled hypertension 47.0% (70/149) Secondary outcome BP screening • Baseline: No hypertension *n* = 185 Did not screen 52.4% (97/185) • Post intervention Did not screen 31.9% (57/185) Reason for not going for screening and starting treatment. Cost (for test and further treatment) and misperceptions were common barriers.	An access-enhanced intervention had some success in improving hypertension management; however, it was less successful in improving cardiovascular risk management, amongst newly diagnosed hypertensives in the rental housing community.	–
Wee LE (2012) [14] Prospective and Qualitative From Jan 2009 to June 2010	Individual and neighbour-hood social factors of hypertension management.	Residents ≥40 y/o. 6 blocks of a socially integrated housing precinct. 3 blocks public rental flats vs 3 blocks of owner-occupied public housing flats. (3 vs 3) Located in Taman Jurong (Western Singapore).	Sample size 710 residents Participation rate 78.9% (710/900) Rental: 90.0% (359/400) VS Owned: 70.2% (351/500) Prevalence rate 64.2 (456/710) Rental: 63.5% (228/359) VS Owned: 65.0% (228/351) Follow up rate: NA Primary outcome • Awareness Rental: 61.8% (141/228) VS Owned: 83.3% (190/228) • On treatment Rental: 69.5% (98/141) VS Owned: 85.3% (162/190) • BP under control Rental: 43.9% (43/98) VS Owned: 66% (107/162) Secondary outcome Independent factors associated with hypertension awareness, treatment and control in rental housing (lower SES) community. • Awareness higher among: Diabetics (adj OR 6.51, CI 2.59–16.37, *p* < 0.001) Dyslipidemics (adj OR 6.74, CI 2.74–16.59, *p* < 0.001) ≥60 years (adj OR 3.08, CI 1.61–5.91, *p* < 0.001) Regular access to a doctor (adj OR 5.63, CI 1.43–22.14, *p* < 0.013). • Treatment more likely among: ≥60 years (adj OR 2.33, CI 1.08–5.01, *p* = 0.031) Treatment less likely among: Need financial aid (adj OR 0.39, CI 0.18–0.83, *p* = 0.016). • Controlled BP less likely among: Employed (adj OR 0.13, CI 0.04–0.41, *p* < 0.001).	Hypertension management (awareness, treatment and control) in those of rental housing community (lower SES) is poorer than in those of owner occupied housing community (higher SES). In rental housing community, awareness was higher among those with diabetes, dyslipidaemia, those ≥60 years and those with regular access to doctors. Treatment was more likely among those ≥60 years, but less likely among those needing financial aid. Control was less likely in the employed.	–
			Qualitative interview: Reasons for not going on hypertensive treatment or participating in regular hypertension screening/monitoring were explored. In the rental housing community: Unknown hypertensives who did not go for regular BP screening in the past 1 year (*n* = 141), the top three reasons: Too busy to go/no time. Testing too expensive. Cost of further treatment, if positive, too expensive. Known hypertensives who were not monitoring their BP regularly (*n* = 64), the top three reasons were similar: Too busy to go/no time. Monitoring too expensive. Cost of further treatment, if positive, too expensive. Reasons for not taking BP medications ≥90% of the time among known hypertensives (*n* = 43): 30.2% (13/43) did not think that the medicine would benefit them. 25.6% (22/43) had problems with the cost of chronic medication. 11.6% (5/43) preferred to take non-Western medication.	Financial barriers need to be addressed for the rental housing community.	
Wee LE (2014) [17] Cross-sectional From Jan to Feb 2012.	Individual and area-level socio-economic status and their association with depression. (GDS-15 ≥ 5)	Residents ≥60 y/o. 2 integrated public housing precinct. Site A (Western Singapore) (3 vs 3) Site B (Eastern Singapore) (7 vs 2)	Sample size 559 residents Participation rate 61.5% (559/909) Site A: 61.3% (236/385) VS Site B: 61.6% (323/524) Rental: 63.7% (398/625) VS Owned: 56.7%, (161/284) (*p* = 0.0473) Prevalence rate 22.9% (128/559) Rental: 26.2% (104/397) VS Owned: 14.8% (24/164) Follow up rate: NA Primary outcome Prevalence rate as above. Secondary outcome Living in a rental housing (lower SES) community was independently associated with depression (adj OR 1.68, CI 1.02–2.84, *p* = 0.049] Not being married (adj OR 2.27, CI 1.35–3.70), falls (adj OR 2.72, CI 1.59–4.67) and poorer social network (adj OR 3.70, CI 1.96–7.14) were associated with depression. Other independent factors associated with depression in rental housing community: Falls (adj OR 2.72, CI 1.59–4.67, *p* < 0.001) Visual impairment (adj OR 2.37, CI 1.28–4.39. *p* = 0.006)	Residing in rental housing was independently associated with depression after controlling for individual SES.	Poor
Wee LE (2012) [18] Cross-sectional From Jan to Feb 2012.	Individual and area Level socio-economic status and Its association with cognitive function and cognitive impairment. (MMSE < 24)	Residents ≥60 y/o. 2 integrated public housing precinct. Site A (Western Singapore) (3 vs 3) Site B (Eastern Singapore) (7 vs 2)	Sample size 558 residents Participation rate 61.4% (558/909) Site A: 61.0% (235/385) VS Site B: 61.6% (323/524) Rental: 63.7% (397/625) VS Owned: 56.7%, (161/284) (*p* = 0.0473) Prevalence rate 23.3% (130/558) Rental 26.2% (104/397) VS Owned 16.1% (26/261) Newly diagnosed in rental housing: 96.2% (100/104) Follow up rate: NA Primary outcome Prevalence rate as above. Secondary outcome Living in a rental housing community was independently associated with cognitive impairment (adj OR 5.13, CI 1.98–13.34, *p* = 0.001).	Living in rental housing is independently associated with cognitive impairment. Many of them with cognitive impairment were undiagnosed prior.	Poor
Wee LE (2016) [19] Cross-sectional From 2009 to 2014 In 2012, a separate study in Site A and Site B focused on those aged ≥60 years old was conducted.	Chronic pain in a low socio-economic status population.	Residents 40–59 y/o. 5 integrated public housing precincts. Site A (Western Singapore) (3 vs 3) Site B (Eastern Singapore) (4 vs 5) Site C (Eastern Singapore) (3 vs 5) Site D (Eastern Singapore) (2 vs 1) Site E (Central Singapore) (2 vs 1)	Sample size 40–59 y/o. 2037 residents ≥ 60 y/o. 559 residents Participation rate 40–59 y/o. Rental: 72.0% (936/1300) VS Owned: 61.2% (1101/1800) ≥ 60 y/o. Rental: 63.7% (397/625) VS Owned: 56.7% (162/284) Prevalence rate 40–59 y/o. Rental: 14.2% (133/936) VS Owned: 14.4% (158/1101) ≥ 60 y/o. Rental: 13.4% (53/397) VS Owned: 13.0% (21/162) Follow up rate: NA Primary outcome Prevalence rate as above. Secondary outcome In the rental housing community, unemployment was associated with chronic pain (adj OR 1.92, 95%, CI 1.05–2.78, *p* = 0.030) Among the elderly, dependency in instrumental activities of daily living (iADLs) was associated with chronic pain (adj OR 2.38, CI 1.11–5.00, *p* = 0.025), as well as female gender, being single, and having higher education (all *p* > 0.05).	There was no difference in pain prevalence between the rental housing community and owner-occupied community. In rental housing community, chronic pain associated with unemployment and functional limitation.	Poor
Wee LE (2017) [20] Cross-sectional and Qualitative From 2009 to 2014	Health screening participation and its association with chronic pain.	Residents 40–59 y/o. 5 integrated public housing precincts. Site A (Western Singapore) (3 vs 3) Site B (Eastern Singapore) (4 vs 5) Site C (Eastern Singapore) (3 vs 5) Site D (Eastern Singapore) (2 vs 1) Site E (Central Singapore) (2 vs 1)	Sample size 40–59 y/o. 2037 residents Participation rate 40–59 y/o. Rental: 72.0% (936/1300) VS Owned: 61.2% (1101/1800) Prevalence rate 40–59 y/o. Rental: 14.2% (133/936) VS Owned: 14.4% (158/1101) Follow up rate: NA Primary outcome In the rental-housing community, chronic pain was associated with higher participation in screening for: Diabetes (adj OR 2.11,CI 1.36–3.27, *p* < 0.001) Dyslipidemia (adj OR 2.06, CI 1.25–3.39, *p* = 0.005) Colorectal cancer (adj OR 2.28,CI 1.18–4.40, *p* = 0.014) Cervical cancer (adj OR 2.65,CI 1.34–5.23, *p* = 0.005) Breast cancer (adj OR 3.52,CI 1.94–6.41, *p* < 0.001) This association was not present in the owner-occupied community. Secondary outcome: NA	Chronic pain was associated with higher cardiovascular and cancer screening participation in the rental housing community.	Poor
			Qualitative interview: General attitudes towards screening tests; and how their pain might affect their attitudes to screening participation. Three main themes emerged from the analysis of the link between chronic pain and screening participation: Pain as an association of “major illness”. Screening as a search for answers to pain. Labelling pain as an end in itself.	To those living in rental housing, disease only occurs when symptoms manifest, such as chronic pain. There is a possibility that chronic pain may present as the “hidden agenda”.	
Health seeking behaviour					
Wee LE (2012) [15] Prospective, Interventional From Jan 2009 to May 2011	Screening for cardio-vascular disease risk factors at baseline and post-intervention.	2 integrated public housing precinct. Site A (Western Singapore) (3 vs 3) Site B (Eastern Singapore) (7 vs 2) Intervention: 6-month community-based intervention comprising access-enhanced screening component and follow-up (outreach) component.	Sample size 1081 residents Participation rate 78.2% (1081/1383) Site A: 83.4% (832/998) VS Site B: 64.6% (249/385). Prevalence rate: NA Follow up rate: NA Primary outcome Baseline screening participation for: Hypertension. Rental 41.7% (150/360) VS Owned 54.1% (139/257) Diabetes. Rental 38.8% (177/456) VS Owned 59.6% (254/426) Dyslipidaemia. Rental 30.8% (128/416) VS Owned 50.2% (165/329) Post intervention: Hypertension. Rental 99.2% (357/360) VS Owned 96.9% (249/257) Diabetes. Rental 45.2% (206/456) VS Owned 67.6% (288/426) Dyslipidaemia. Rental 37.0% (154/416) VS Owned 58.4% (192/329) Secondary outcome Living in a rental housing community (adj RR 0.61, CI 0.37–0.99, *p* = 0.048) and having hypertension (adj RR 0.45, CI 0.18–0.98, *p* = 0.049) was associated with lower participation in screening for diabetes and dyslipidaemia respectively. Employment (adj RR 1.57, CI 1.03–2.60, *p* = 0.040) and Chinese ethnicity (adj RR 1.84, CI 1.00–3.43, *p* = 0.050) was associated with higher participation in screening for diabetes and dyslipidaemia respectively.	Those living in rental housings had lower participation in screening before intervention, when compared against those living in owner-occupied housings. Post intervention, participation rates for all three screening modalities rose significantly by similar proportions in both rental and owner-occupied community (all *p* < 0.001).	–
Wee LE (2012) [16] Prospective, Interventional From Jan 2009 to May 2011	Socio-economic factors affecting colorectal, breast and cervical cancer screening at baseline and post-intervention.	2 integrated public housing precinct. Site A (Western Singapore) (3 vs 3) Site B (Eastern Singapore) (7 vs 2) Intervention: 6-month community-based intervention comprising access-enhanced screening component and follow-up (outreach) component.	Sample size 1081 residents Participation rate 78.2% (1081/1383) Site A: 83.4% (832/998) VS Site B: 64.6% (249/385). Prevalence rate: NA Follow up rate: NA Primary outcome Baseline screening participation for: Colorectal cancer. Rental 7.7% (33/427) VS Owned 16.6% (66/397) Cervical cancer. Rental 20.4% (44/216) VS Owned 41.9% (93/222) Breast cancer. Rental 14.3% (46/321) VS Owned 15.9% (48/302) Post intervention: Colorectal cancer. Rental 19.0% (81/427) VS Owned 28.2% (112/397) Cervical cancer. Rental 25.4% (55/216) VS Owned 47.3% (105/222) Breast cancer. Rental 17.4% (56/321) VS Owned 16.9% (51/302) Secondary outcome Factors associated with cancer screening in rental housing community: Males (adj OR 2.11, CI 1.01–4.42) and those overweight (adj OR 2.76, CI 1.32–5.75) was associated with higher participation in colorectal cancer screening. The employed (adj OR 1.56, CI 1.03–2.35) and those of higher educational status (adj OR 1.96, CI 1.27–3.02) was associated with higher participation in breast cancer screening. Cost was a major factor in the low-SES community, especially for pap smears/mammograms. Misperceptions and lack of time/awareness were also important.	Those living in rental housings had lower participation in colorectal and cervical cancer screening before intervention, when compared against those living in owner-occupied housings. Post intervention, participation rates rose for most screening modalities in both communities (all *p* ≤ 0.001), except for breast cancer in the owner-occupied community. (*p* = 0.250).	–
Ng CW (2012) [21] Cross-sectional From Jun to Oct 2009	Characteristic associated with non-willingness to participate in health promotion programmes	Residents ≥18 y/o. 4 blocks of 1 to 2 rooms housing estate. Located in Toa Payoh.	Sample size 974 residents Participation rate 79.9% (778/974) Prevalence rate: NA Follow up rate: NA Primary outcome 36.1% (281/778) of residents were willing to participate in at least one health promotion programme (health screening, talk or workshop). Older residents aged 45–64 years (OR 0.52, CI 0.35–0.76, *p* = 0.001) and more than 65 years (OR 0.44, CI 0.29–0.66, *p* < 0.001) were less likely to participate than their younger counterparts (18–44 years). Malays (OR 1.84, CI 1.27–2.68, *p* = 0.001) were more likely than Chinese to participate, and residents who do not exercise (OR 0.57, CI 0.42–0.78, *p* < 0.001) were less likely to participate than residents who exercise (regularly/occasionally). Secondary outcome Reasons for non-willingness to participate were ‘not interested’ and ‘no time’.	Residents living in 1 to 2 room housing had low rate of participation in health promotion programme. Older residents and those who do not exercise had lower rate of participation as well.	Poor
Wee LE (2011) [22] Cross-sectional, Interventional From Jan 2009 to May 2010	The effect of neighbour-hood socio-economic status and a community-based program on multi-disease health screening.	Residents ≥40 y/o. 6 blocks of a socially integrated housing precinct. (3 vs 3) Located in Taman Jurong (Western Singapore). Intervention: 6-month community-based intervention comprising access-enhanced screening component and follow-up (outreach) component.	Sample size 707 residents Participation rate 78.6% (707/900) Rental: 89.0% (356/400) VS Owned: 70.2% (351/500) Prevalence rate: NA Follow up rate: NA Primary outcome Baseline screening participation for: Hypertension. Rental 35.8% (77/215) VS Owned 52.2% (84/161) Diabetes. Rental 35.0% (98/280) VS Owned 66.0% (190/288) Dyslipidaemia. Rental 26.2% (70/267) VS Owned 53.1% (119/224) Colorectal cancer. Rental 6.0% (15/251) VS Owned 17.0% (49/288) Post intervention: Hypertension. Rental 98.6% (212/215) VS Owned 100.0% (161/161) Diabetes. Rental 40.0% (112/280) VS Owned 66.7% (192/288) Dyslipidaemia. Rental 30.3% (81/267) VS Owned 54.0% (121/224) Colorectal cancer. Rental 16.3% (41/251) VS Owned 18.7% (54/288) Secondary outcome Living in a better-off neighbourhood was independently associated with diabetes mellitus (66% vs. 35%, adj OR 2.12, *p* < 0.01), hyperlipidemia (53% vs. 26%, adj OR 4.34, *p* < 0.01) and colorectal cancer screening (17% vs. 6%, adj OR 15.43, *p* < 0.01), as were individual socioeconomic factors such as employment, need for financial aid and household income. Cost was cited more commonly as a barrier to health screening in the rental housing community. Reasons for not participating in screening in both community, and for a majority of modalities: Misperceptions Lack of time	Uptake of all screening modalities significantly increased in the rental housing community post-intervention (all *p* < 0.05).	Poor
Wee LE (2016) [23] Cross-sectional and Qualitative From 2009 to 2014	Primary care characteristic and their association with health screening.	Residents 40–59 y/o. 5 integrated public housing precincts. Site A (Western Singapore) (3 vs 3) Site B (Eastern Singapore) (4 vs 5) Site C (Eastern Singapore) (3 vs 5) Site D (Eastern Singapore) (2 vs 1) Site E (Central Singapore) (2 vs 1)	Sample size 1996 residents Participation rate 64.4% (1996/3100) Rental: 72.0% (936/1300) VS Owned: 58.9% (1060/1800) Prevalence rate: NA Follow up rate: NA Primary outcome Rental: Regular primary care was independently associated with regular: Diabetes screening (adj OR 1.59, CI 1.12–2.26, *p* = 0.009). Hyperlipidemia screening (adj OR 1.82, CI 1.10–3.04, *p* = 0.023). Proximity to primary care was associated with less participation in regular: Colorectal cancer screening (adj OR 0.42, CI 0.17–0.99, *p* = 0.049) Breast cancer screening (adj OR = 0.29, CI 0.10–0.84, *p* = 0.023). Usage of subsidized primary care was only associated with increased participation in regular: Breast cancer screening (adj OR 2.33, CI 1.23–4.41, *p* = 0.009). Owned: Regular primary care was independently associated with regular: Hypertension screening (adj OR 9.34 CI 1.82–47.85, *p* = 0.007) Usage of subsidized primary care was associated with regular: Diabetes screening (adj OR 2.94, CI 1.04–8.31, *p* = 0.042). Proximity to primary care was associated with higher participation in regular: Colorectal cancer screening (adj OR 1.48, CI 1.01–2.21, *p* = 0.049). Usage of subsidized primary care was associated with higher participation in regular: Cervical cancer screening (adj OR 7.93. CI 1.03–62.51, *p* = 0.047) Breast cancer screening (adj OR 6.02, CI 1.69–21.28), *p* = 0.006) Proximity to primary care was associated with higher participation in regular: Cervical cancer screening (adj OR 3.22, CI 1.72–5.84, *p* < 0.001) Breast cancer screening (adj OR 2.22, CI 1.08–4.54), *p* = 0.032) Regular primary care follow up was associated with less participation in regular: Breast cancer screening (adj OR 0.10, CI 0.01–0.75, *p* = 0.025). Secondary outcome: NA	Regular primary care was independently associated with regular participation in cardiovascular screening in both rental housing and owner occupied communities. However, for cancer screening, in the rental housing community, proximity to primary care was associated with less participation in regular screening, while in the owner occupied housing community, regular primary care was associated with lower screening participation; possibly due to embarrassment regarding screening modalities.	Poor
			Qualitative interview: To elicit perceptions about cardiovascular disease and cancer screening. Major themes and subthemes: • Primary care characteristics (Barriers) Lack of trust in healthcare system/healthcare professionals Healthcare professional does not often discuss screening – no time Embarrassment about screening modality Characteristics of clinic (manpower, location, hours open) • Knowledge (Barriers) Not aware of screening No need screening as healthy /not at risk Not aware of where to go for screening Screening may not be accurate/alternative screening methods are better Last test normal, so no need to go again Confusion that mammogram causes cancer • Priorities (Barriers) No time to go, too busy Can spend money on other things • Attitudes (Barriers) Fatalism Fear of diagnosis and/or treatment Too old to go for screening Traditional medicine is better Disease not important	Patients were discouraged from screening by distrust in the doctor-patient relationship; for cancer screening in particular, patients were discouraged by potential embarrassment.	
Healthcare utilisation					
Wee LE (2014) [24] Cross-sectional and Qualitative From Jan 2009 to May 2010	Choice of primary health care source.	Residents ≥40 y/o. 6 blocks of a socially integrated housing precinct. (3 vs 3)	Sample size 710 Participation rate 88.6% (710/800) Rental: 89.8% (359/400) VS Owned: 87.8% (351/400) Prevalence rate: NA Follow up rate: NA Primary outcome Preferred source of medical treatment and advice Rental: Rely on own knowledge. 52.6% (189/359) Alternative medicine practitioners. 29.5% (106/359) Family/friends. 6.7% (24/359) Western-trained doctors. 11.1% (40/359) Owned: Rely on own knowledge. 54.1% (190/351) Alternative medicine practitioners. 2.0% (7/351) Family/friends. 14.0% (49/351) Western-trained doctors. 29.9% (105/351) Secondary outcome Residents staying in rental housing (compared with those staying in owner-occupied housing) were less likely: to seek advice from Western-trained doctors (adj OR 0.36, CI 0.21–0.61, *p* < 0.001) to seek advice from family members (adj OR 0.36, CI 0.19–0.69, *p* < 0.002) They were more likely: to turn to alternative medicine practitioners (adj OR 14.29, CI 4.55–50.00, *p* < 0.001) In rental housing community: Unmarried were more likely to consult alternative medicine practitioners (adj OR 3.13, CI 1.41–6.67, *p* = 0.005) Minority ethnicity were more likely to consult family members (adj OR 3.23, CI 1.23–8.33, *p* = 0.016) Higher household income (≥$500/month) were less likely to seek consult from anyone, relying instead on their own knowledge (85.2%, 161/359) With dyslipidemia were less likely to consult alternative medicine practitioners (adj OR 0.34, CI 0.14–0.83, *p* = 0.017).	Western-trained physicians are not the first choice of seeking primary care in the rental housing community.	Poor
			Qualitative interviews: To elicit perspectives on barriers/enablers that lower income patients face in seeing a Western-trained physician for primary care. Patient and provider comments fell into the following content areas: Primary care characteristics- trust, distance, waiting time. Knowledge- healthy, not effective, minor ailment. Costs-for treatment, subsidies. Priorities- busy Attitudes- fear of diagnosis and treatment Information sources- Media (TV, newspaper) Self-reliance was perceived as acceptable for ‘small’ illnesses but not for ‘big’ ones. Communal spirit was cited as a reason for consulting family/friends. Social distance from primary care practitioners was highlighted as a reason for not consulting Western-trained doctors.	Knowledge, primary care characteristics and costs were identified as Potential barriers/enablers.	
Low LL (2016) [25] Retrospective cohort From Jan 2014 to Dec 2014	Housing as a social determinant of health and its association with readmission risk and increased utilisation of hospital services.	Patients who have at least one clinical encounter (admission or ED visit) to Singapore General Hospital (SGH) in 2014. Patients, who died in 2014, are non-residents, who resided in areas where SGH is not the primary hospital or patients discharged to long-term residential care facilities were excluded.	Sample size A total of 14,457 patients were analyzed and 2163 patients (15.0%) were rental housing residents. Primary outcome Readmission within 15 days associated with residence in public rental housing: OR 1.19, CI 1.02–1.39, *p* = 0.029 Readmission within 30 days: OR 1.27, CI 1.12–1.43, *p* < 0.001 Frequent hospital admissions: OR 1.27, CI 1.14–1.43, *p* < 0.001 Frequent ED attendances: OR 1.40, CI 1.21–1.61, *p* < 0.001, Staying in public rental housing showed a 8% lower risk per one SOC visit, but the result was statistically non-significant, 0.92 (0.83–1.02), *p* = 0.112 Secondary outcome: NA	Patients staying in rental housings have a 19 and 27% higher odds of being readmitted within 15 and 30 days, respectively. Patients staying in rental housings have a 27 and 40% higher risk of being a frequent hospital admitter and frequent ED attendee, respectively.	Fair

**Table 2:** List of variables for data extraction of included studies

Health status	
1	Mortality
2	Diseases prevalence rate
	Diseases incidence rate
3	Diseases management • Being treated, on treatment • Well controlled
Health seeking behaviour	
4	Participation in Health screening • Chronic disease: Hypertension, Hyperlipidaemia, Diabetes • Cancer screening: Breast cancer, Cervical cancer, Colorectal cancer
5	Participation in Health promotion programme
Healthcare utilisation	
6	Utilisation of primary and community care • Primary care services • Home care services e.g. home nursing, home medical services
7	Utilisation of hospital care Hospital admission • Emergency department attendances • Hospital clinics

Including but not limited to

Socio-demographic characteristics of the population were collected in all the articles. There was a larger proportion of elderly living in the rental housing. More than half of them were single and not married. There was an almost equal distribution of both genders. Most of them had no formal education or only primary school education [17]. Singapore is a multi-ethnic urbanised Asian society, there is an ethnic integration policy in place to maintain a good ethnic mix in our public housing estate (Housing Development board, HDB), thereby helping to promote racial integration and harmony. However, there was a slightly higher percentage of non-Chinese staying in the rental housing as compared to owner-occupied housings [17–19]. Rental housing are heavily subsidised housing for those who have low or no household income and have no assets. They were mainly elderly, unemployed and on financial aid for healthcare or daily living.

Among the 14 articles, seven of them mainly covered the outcome related to one’s health status, five articles on their health seeking behaviour and the last two were on the healthcare utilization.

### Health status

Health status was being studied in many aspects in these articles, ranging from different diseases namely head and neck carcinoma, hypertension, depression, cognitive impairment and chronic pain. Different outcomes of health status were examined as well like mortality, prevalence of disease and various association factors with some of these being compared between the rental housing and owner-occupied housing community.

There was only one study done to find out if a patient’s housing type influenced mortality [26]. In those with head and neck squamous cell carcinoma, it was found that those staying in a smaller size, rental housing community (11% of the total patients analysed) had poorer survival [median, 28 months, CI 21–48 months] compared to those staying in larger housing sizes [median 42 months, CI 24–65 months] despite no apparent delays in presentation.

We found the prevalence of depression [17] and cognitive impairment [18] to be higher in the rental housing community as compared to the owner-occupied housing community (depression 26.2 vs 14.8% and cognitive impairment 26.2 vs 16.1%). Whereas, the prevalence of hypertension [14] and chronic pain [19] was similar between rental housing community and owner-occupied housing community (hypertension 63.5 vs 65.0% and chronic pain 13.4 vs 13.0%). The prevalence of hypertension and chronic pain in the studies were actually higher than the national estimates. Hypertension prevalence rate was 64.2 while estimate from National Health Survey 2010 was 23.5%. Chronic pain prevalence rate was 13.4%, as compared to local population-wide estimates of 8.7%.

More than half of the diagnosed hypertension cases were untreated (53.5%) and uncontrolled (54.2%) despite on treatment. A 6-month community-based intervention improved hypertension management but not significantly for the screening of hypertension and additional cardiovascular risk screening. In addition to having higher numbers of untreated and uncontrolled hypertension, rental housing residents had poorer awareness of their disease. From the qualitative interviews, the reasons for poor hypertension management were mainly being busy and lack of time for care. Another common cited reason was cost of screening and treatment.

It was also found that medical comorbidities such as falls [adjusted (adj) OR 2.72, CI 1.59–4.67, *p* < 0.001] and visual impairment (adj OR 2.37, CI 1.28–4.39, *p* = 0.006) were independently associated with depression. Being married (adj OR 0.44, CI 0.27–0.74, *p* = 0.002) and having larger social networks (adj OR 0.27, CI 0.14–0.51, *p* < 0.001) were protective factors against depression.

Staying in a rental housing was found to be independently associated with cognitive impairment (adj OR 5.13, CI 1.98–13.34, *p* = 0.001) and many (96.2%) had cognitive impairment that was newly diagnosed only after the screening done during the study.

There was an association between chronic pain with unemployment (adj OR 1.92, CI 1.05–2.78, *p* = 0.030) and being less independent in instrumental activities of daily living (adj OR 0.42, CI 0.20–0.90, *p* = 0.025). In another study on chronic pain [20], it was found that those with chronic pain had higher participation in screening for diabetes (adj OR 2.11, CI 1.36–3.27, *p* < 0.001), dyslipidaemia (adj OR 2.06, CI 1.25–3.39, *P* = 0.005), colorectal cancer (adj OR 2.28, CI 1.18–4.40, *p* = 0.014), cervical cancer (adj OR 2.65, CI 1.34–5.23, *p* = 0.005) and breast cancer (adj OR 3.52, CI 1.94–6.41, *p* < 0.001). And this was not seen in the owner-occupied housing community. There was a qualitative interview in this study that explored the general attitudes towards screening tests and how their pain might affect their attitudes to screening participation. Three main themes emerged from the analysis of the link between chronic pain and screening participation was: pain was identified as an association of “major illness”, screening was as a search for answers to pain and labelling pain as an end in itself.

### Health seeking behaviour

Many people may fail to go for health screening and may ignore minor symptoms resulting in delayed treatment. Participation in a health screening is reflection of a person’s health seeking behaviour. Four studies concentrated mainly on cardiovascular risk factor and cancer screening, including 1 that explored if primary care characteristic had any association with health screening in the rental housing community. The 5th study evaluated willingness for health promotion programme participation.

For these studies [15, 16, 22], there was a comparison of the screening participation rate between the rental housing and owner-occupied housing community. At the same time, intervention which included a screening and follow-up component was done and the change in health screening uptake rate was monitored. For cardiovascular risk factors screening, those staying in rental housing had much lower participation rate [Hypertension, 41.7% (rental) vs 54.1% (owned), Diabetes 38.8 vs 59.6%, Dyslipidaemia, 30.8 vs 50.2%]. Cancer screening participation rate was also lower in the rental housing community (colorectal cancer 7.7 vs 16.6%, cervical cancer 20.4 vs 41.9%) except for breast cancer screening with not much difference (14.3 vs 15.9%) between the two communities.

Participation rates had increased for most of the screening modalities after intervention, however it was noted that breast cancer screening participation rate rose the least even in the owner-occupied housing community. More commonly cited barrier to health screening was concern about cost in the rental housing community [15, 16].

Other reasons for not participating in screening were lack of time, misperceptions about screening (for example, they may feel that they were healthy or not at risk, hence it was not necessary) and lack of interest [21].

In the study that explored on the primary care characteristic association with health screening [23], seeing a regular primary care doctor was independently associated with regular diabetes and hyperlipidaemia screening. There was less participation in regular colorectal cancer screening and breast cancer screening with proximity to primary care. Lastly, with subsidised primary care, there was associated increased for participation in regular breast cancer screening.

Qualitative interview section elicited perceptions from the residents of rental housing about cardiovascular risk factors and cancer screening. The major themes were barriers related to the primary care characteristics, the residents’ knowledge, priorities and attitudes. For primary care characteristics, lack of trust in the healthcare system or healthcare professionals; lack of time from healthcare professionals to discuss about screening and the embarrassment associated with screening modality like PAP smear for cervical cancer. Characteristics of clinic such as manpower, location and opening hours were cited as barriers in seeking for health screening. As to the barriers in knowledge, it was found that many were not aware of health screening; felt that there was no need for screening as they were healthy and therefore not at risk and lack of awareness of where to go for screening. Some felt that screening may not be accurate and alternative screening methods were better; their previous test was normal with no need to repeat screening and there was misperception that mammogram caused cancer. Lack of time and cost were re-iterated as barriers to health screening, while fatalism attitudes and old age were likewise raised. Some had the fear of diagnosis and/or treatment with others who believed that traditional medicine was better.

### Healthcare utilisation

SES and perception may influence the way patients utilised health care services. The last two studies focused on the choice of primary health care source [24] in the rental housing community and their utilisation of hospital services [25] respectively.

Rental housing residents relied on their own knowledge (52.6%) before seeking medical treatment and advice. More preferred alternative medicine practitioners (29.5%) to western-trained doctors in the primary care (11.1%). There was about 6.7% of them relied on their family/friends. On the other hand, seeking help from alternative medicine practitioners was the least preferred source in the owner-occupied housing community. It was also noted that among rental housing community, those who consult alternative medicine practitioners were more likely not married and those of minority ethnicity were more likely to consult their family members.

Qualitative interviews were carried out to elicit the perspectives on barriers/enablers that they faced in seeing western-trained doctors in primary care. The views were from both the patients and providers with their comments as per following content areas: primary care characteristics like waiting time, knowledge in terms of perception as minor ailment, costs of treatment, priorities, attitudes like fear of diagnosis and lastly depending on their information sources. ‘Small’ illnesses were perceived as acceptable as part of self-reliance but not for ‘big’ illnesses. Having the communal spirit was the reason for consulting family/friends. An interesting fact about having social distance from primary care doctors was highlighted as a reason for not consulting western-trained doctors.

Staying in public rental housing was an independent risk factor for readmission, frequent hospital admissions and ED attendances in Singapore [25]. The consistent trend of the outcomes showed that there was a strong, consistent link between staying in public rental housing with an increased in hospital utilisation.

## Discussion

While there has been numerous studies evaluating the association between staying in public rental housing and health, this is the first review to summarise these relationships with health (health status, health seeking behaviour and healthcare utilisation).

Singapore is a small, multi-ethnic Asian country undergoing rapid urbanisation. Urban planning in Singapore takes into consideration the need to prevent development of disadvantaged neighbourhoods [27]. The Urban Redevelopment Authority of the country is well known for its meticulous planning with regards to population distribution and the allocation of public amenities. This includes ensuring that all residents have ready access to healthcare facilities. Notwithstanding that, in this review, it was found that residents of public rental housings have poorer health status and outcome. These residents had lower participation in health screening, preferred alternative medicine practitioners to western-trained doctors in primary care. Lastly, hospital utilisation was increased among them. Traditionally, studies had found that different individual markers of SES such as employment status, educational level and housing type are associated with poorer health [28, 29]. Compared to markers such as employment status and educational level, housing type is part of the patient’s address and is easily retrievable from the electronic health record. In Singapore, residents in the same apartment block share the same postal code. In the on-going effort to improve health and living conditions of the economically disadvantaged, policy makers can use this as a proxy marker of SES [30, 31] to identify high-risk populations and direct intervention programs to where it is most needed.

**Fig. 2:**
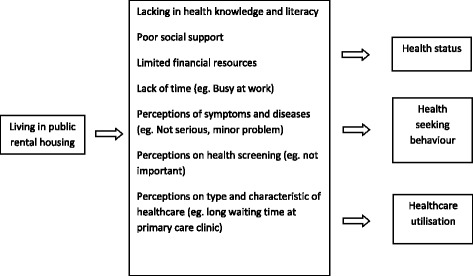
Mechanism map of the association between living in public rental housing with poorer health

This findings in this review provides good insights into possible reasons for poorer health of residents of public rental housings in Singapore. We had developed a mechanism map to explained the association between living in public rental housing with poorer health (Fig. 2). Firstly, perceptions and health literacy have a major impact on health. The elderly staying in the rental housing tend not to be highly educated [18] and had poor health literacy. For example, many had low awareness and misperceptions about cognitive impairment or dementia, accepting it as part of normal ageing. Studies that intervened to increase participation rate in health screening were unsuccessful despite making it free of charge and providing it at the residents’ doorstep at a time of their choice. A possible reason may be that such elderly persons are averse to discovering additional health problems when they are already having difficulties coping with existing problems of living. A different approach such as reassuring the participants of the availability of additional support for them when health issues are discovered may prove to be more effective.

Secondly, the juxtaposition of low SES apartment blocks within communities of higher SES may increase perception of inequality, a lack of social support and social distance. Local social inequality [7] may have negative impact on the health status of residents of public rental housings. Wide disparities in income may result in diminished trust in the community that could lead to social withdrawal. Such social isolation may have negative effects on health especially on cognition and depression. The sense of community would be low when one stay in a high-rise apartment where communal interaction are limited to immediate neighbours due to the shortage of community space. This is exacerbated in elderly staying in rental housing, who had smaller social network and are mostly living alone. One study found that elderly persons with a weak social network are more likely to suffer from pain and the progression of chronic pain [32]. For cancer patients and those with disabilities, access to amenities in the community and to public transport is critical especially when they have to commute regularly for visits or treatment at healthcare facilities. Consideration to these areas should be given when developing policies or services for patients of low SES. Effort must be made to create opportunities for social interaction between residents in high rise apartment blocks. Another example of social distance hindering health status was the lack of trust between the doctor and the patient when the latter felt that doctors are not in touch with the reality of staying in the public rental housing. This could be improved but it requires investment in building a good doctor-patient relationship and ensuring continuity of care [33].

Thirdly, the anxiety over the affordability of medical care among residents of public rental housings deter many of them from seeking medical help especially in the screening of chronic disease. In Singapore, one of the recent new interventions to improve access to healthcare for such patients was the Community Health Assist Scheme (CHAS). It provides additional subsidy to Singaporeans with lower household income for primary care. Recipients of this assistance scheme can seek treatment from private General Practitioners (GP) who are often located within communities where such rental housings are found. At the time of writing there were about 1650 GPs in private practice who have signed up for the CHAS since its inception in 2012. About 1.3 million Singaporeans are eligible for this scheme [34]. More of such targeted subsidy schemes may give elderly persons with low SES the confidence to take up health screening programs and adhere to the management plans of their chronic diseases.

### Limitation

Eleven out of the 14 studies used for the review were done by the same author throughout several years at a maximum of five integrated public housing precincts that may not be nationally representative of public rental housing communities. Therefore, the results may not be fully generalisable to all the rental housing population Singapore.

However, rental housing applicants are randomly allocated to available housings within the different geographical zones. Therefore, the demographics should be similar across the different rental housing communities in Singapore.

For the study on hospital services utilisation [25], readmissions to other health systems was not accounted for. In order to reduce bias, patients who stay in geographical locations served by other health systems were not included in the study.

Overall, the 14 appraised articles were mainly of cross-sectional and retrospective cohort study designs with poor quality of evidence. For the interventional studies, the follow-up period was relatively short over a year. Lost to follow-up may have led to selection bias. Those who were more likely to continue participation in the study may have better diseases management.

In the cross-sectional studies, there would be response bias; those who declined to participate may reject to participate due to presence of condition such as depression. Therefore, it is possible that the prevalence of depression in the study may be underestimated. Moreover, causality cannot be inferred. Future studies may validate self-reported chronic diseases with hospital and clinic records to determine the true prevalence of chronic diseases and psychological conditions.

### Direction of future research

Studies can be conducted in a randomly selected, nationally representative sample of public rental housing residents in Singapore to provide generalisable data.

Targeted interventional research studies directed at the rental housing community may be carried out to address the health inequalities. More qualitative studies can be done to interview the residents staying in rental housing to further explore on their health seeking behaviour and health literacy. Such findings can be used to inform and guide the interventional studies as they may be more unknown entities with regards to the gaps of current services provided to the community.

### Conclusion

Our review provides an important summary of the evidence on the association of public rental housing with poor health status, lower participation in health screening and higher hospital utilisation but under-utilisation in primary care. These findings have important public health implications for health and housing policy planners in Singapore. Future studies should be conducted in a nationally representative public rental housing cohort and should include qualitative studies to obtain a deeper understanding of the social circumstances, health seeking behaviour and their impact on health in these communities.
